# Lysyl Oxidase (LOX) Family Proteins: Key Players in Breast Cancer Occurrence and Progression

**DOI:** 10.7150/jca.98688

**Published:** 2024-08-13

**Authors:** Jinsong Li, Xinmeng Wang, Ruai Liu, Jiaji Zi, Yihan Li, Zhengliang Li, Wei Xiong

**Affiliations:** 1 Department of Biochemistry and Molecular Biology, College of Basic Medical Sciences, Dali University, Dali 671000, Yunnan, China.; 2 Key Laboratory of Clinical Biochemistry Testing in Universities of Yunnan Province, College of Basic Medical Sciences, Dali University, Dali 671000, Yunnan, China.; 3 Yunnan Provincial Key Laboratory of Entomological Biopharmaceutical R&D, College of Pharmacy, Dali University, Dali 671000, Yunnan, China.; 4 Department of Radiology, The First Affiliated Hospital of Dali University, Dali University, Dali 671000, Yunnan, China.

**Keywords:** lysyl oxidase (LOX), family proteins, extracellular matrix (ECM), breast cancer, invasion and metastasis, prognosis

## Abstract

The lysyl oxidase (LOX) family proteins are secreted copper-dependent amine oxidases, comprised of five paralogues: LOX and LOX-like 1-4 (LOXL1-4), which are characterized by catalytic activity contributing to the remodeling of the cross-linking of the structural extracellular matrix (ECM). ECM remodeling plays a key role in the angiogenesis surrounding tumours, whereby a corrupt tumour microenvironment (TME) takes shape. Additionally, dysregulation and aberrant expression of LOX family proteins have been implicated in the occurrence and progression of various types of human cancers, including lung cancer, hepatocellular carcinoma, gastric cancer, renal cell carcinoma, and colorectal cancer. Breast cancer is the most prevalent malignant tumour in women worldwide, and its incidence rate is increasing annually. In recent years, a growing body of evidence has revealed significant upregulation of LOX family proteins in breast cancer, which contributes to cancer cell proliferation, invasion, and metastasis. Furthermore, elevated expression of LOX family proteins is closely associated with poor prognosis in breast cancer patients. We herein review the structure, regulation, function, and mechanisms of LOX family proteins in the occurrence and progression of breast cancer. In addition, we highlight recent insights into their mechanisms and their potential involvement in the clinical value and novel biological roles of LOX family members in tumour progression and the TME of breast cancer. This review will provide a theoretical basis and reference for clinical diagnosis and treatment of breast cancer, as well as for the screening of effective LOX-specific inhibitors.

## Introduction

Breast cancer is the most prevalent malignant tumour in women worldwide, and its incidence rate is increasing annually 1. The mortality rate associated with breast cancer is notably high due to the dissemination of malignant tumour cells from the primary site to distant areas of the body. Despite advancements in early detection and treatment, managing metastasis in breast cancer patients remains a significant clinical challenge. Metastasis commonly occurs in bones, lungs, liver, and brain, leading to increased patient mortality rates 2. Approximately 1/3 of breast cancer patients who receive local treatment eventually experience distant metastatic recurrence, which significantly impacts patient prognosis. Tumour-stroma interactions, as well as remodelling and alterations of the extracellular matrix (ECM), are implicated in the progression and metastasis of breast cancer 3. Lysyl oxidase (LOX) family proteins, including LOX and four lysyl oxidase-like proteins (LOXL1-LOXL4), are copper-dependent amine oxidases that facilitate the oxidative deamination of lysine residues on collagen and elastin within the ECM. This process results in the formation of intramolecular and intermolecular covalent cross-links, which play a crucial role in ECM formation and repair 4. Recent research has highlighted a strong association between abnormal expression of LOX family proteins and the proliferation, migration, invasion, and metastasis of breast cancer cells 5. LOX and LOXL1-LOXL4 have emerged as potential therapeutic targets for inhibiting breast cancer metastasis. This review focuses on elucidating the structure, regulation, function, and mechanisms of action of LOX family proteins in breast cancer, offering valuable insights for clinical diagnosis and the development of innovative treatment approaches for this disease.

## Overview of LOX family proteins

### Structure of LOX family proteins

**Figure 1:**
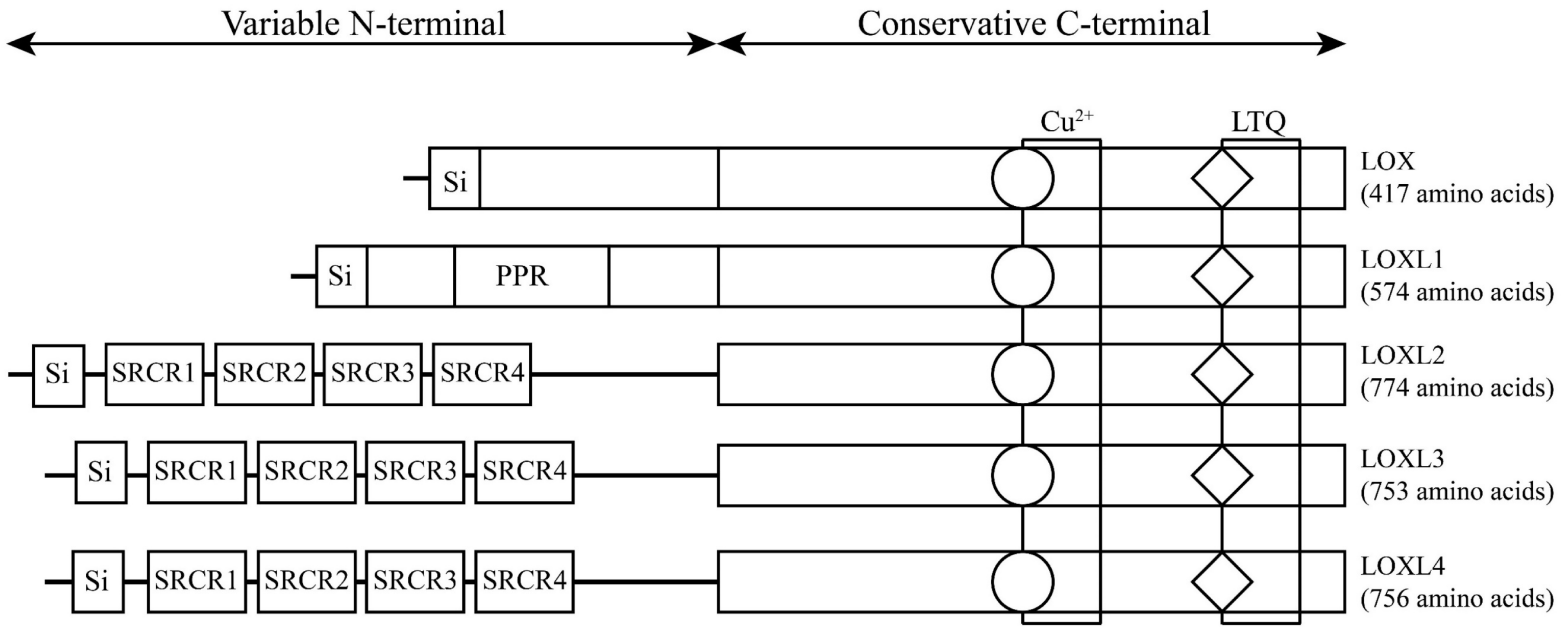
The structure of lysyl oxidase (LOX) family proteins. LOX family members encoded by the human LOX/LOXLs genes are located at various chromosome sites, including 5q23.1, 15q24.1, 8p21.3, 2p13.1, and 10q24.2. These members consist of a variable N-terminal domain and a highly conserved C-terminal domain. Si, signal peptide (Si); scavenger receptor cysteine-rich (SRCR); proline-rich region (PPR); copper binding domain (Cu^2+^); lysyl-tyrosyl-quinone (LTQ) co-factor; cytokine receptor-like domain (CRL).

The LOX family, which comprises five members, is encoded by the LOX/LOXL genes located on specific chromosomal regions in humans, namely, 5q23.1, 15q24.1, 8p21.3, 2p13.1, and 10q24.2 6 (**Table 1**). These genes give rise to proteins with a distinct structural composition characterized by a conserved C-terminal domain and a variable N-terminal domain. The conserved C-terminal domain features copper ion-binding sites, lysine tyrosylquinone (LTQ) residues, and cytokine receptor-like (CRL) domains. The N-terminal regions of LOX and LOXL1 contain a proline-rich region (PRR) and signal peptide (Si) sequences, while LOXL2, LOXL3, and LOXL4 have four scavenger receptor cysteine-rich domains (SRCRs) in their N-terminal regions 7 (**Figure 1**). This structural diversity highlights the unique functional attributes of each member of the LOX family, underscoring their potential involvement in various biological processes. LOX and LOXL1 activation requires a specific cleavage process facilitated by extracellular bone morphogenetic protein 1 (BMP-1), whereas LOXL2, LOXL3 and LOXL4 do not rely on this process for their activation and maturation. Initially, LOX mRNA is translated into an inactive LOX proprotein (pro-LOX) within the cytoplasm. Subsequently, pro-LOX is secreted from the cell, and the N-terminus of the protein undergoes cleavage by BMP-1, resulting in the generation of the LOX propeptide (LOX-PP) and the mature, active LOX protein, which then perform their respective functions 8.

### Regulation of the LOX family proteins

Limited research has been conducted on the regulatory mechanisms controlling the expression of LOX family proteins. Recent investigations have identified several key modulators, including hypoxia-inducible factors (HIFs), transforming growth factor β (TGF-β), forkhead box transcription factor family M1b (FOXM1b), and microRNAs (miRNAs).

Hypoxia has been recognized as a crucial regulatory factor for the LOX family. For instance, in breast cancer and head and neck tumour cells, hypoxia-induced HIF-1α interacts with the hypoxia-inducible element (HRE) of LOX, thereby enhancing the transcription and expression of LOX. Moreover, studies have suggested that LOX is particularly sensitive to regulation by HIF-2α 9. In breast cancer cells, TGF-β activates signalling pathways, such as the Smad3, PI3K, and MAPK pathways, leading to a dose- and time-dependent increase in LOX mRNA expression 10. In hepatocellular carcinoma, the FOXM1b transcription factor has been shown to directly bind to the LOX and LOXL2 genes promoters, thereby facilitating their expression 11. Conversely, in breast cancer, the GATA-3 transcription factor acts as a negative regulator by promoting the methylation of the LOX promoter, consequently reducing LOX expression 12. Noncoding RNAs also play a role in the regulation of LOX. For example, miR-30a targets LOX to impede the progression of undifferentiated thyroid carcinoma 13. In idiopathic pulmonary fibrosis and lung cancer, miR-29a suppresses the expression of LOXL2 14. Similarly, in prostate cancer, miR-26a, miR-26b, miR-29a, miR-29b, miR-29c, and miR-218 have been identified as inhibitors of LOXL2 expression 15. However, studies on the regulation of LOXL1, LOXL3, and LOXL4 by noncoding RNAs are still lacking.

## LOX family expression and mechanism in breast cancer

### LOX family expression in breast cancer

**Figure 2:**
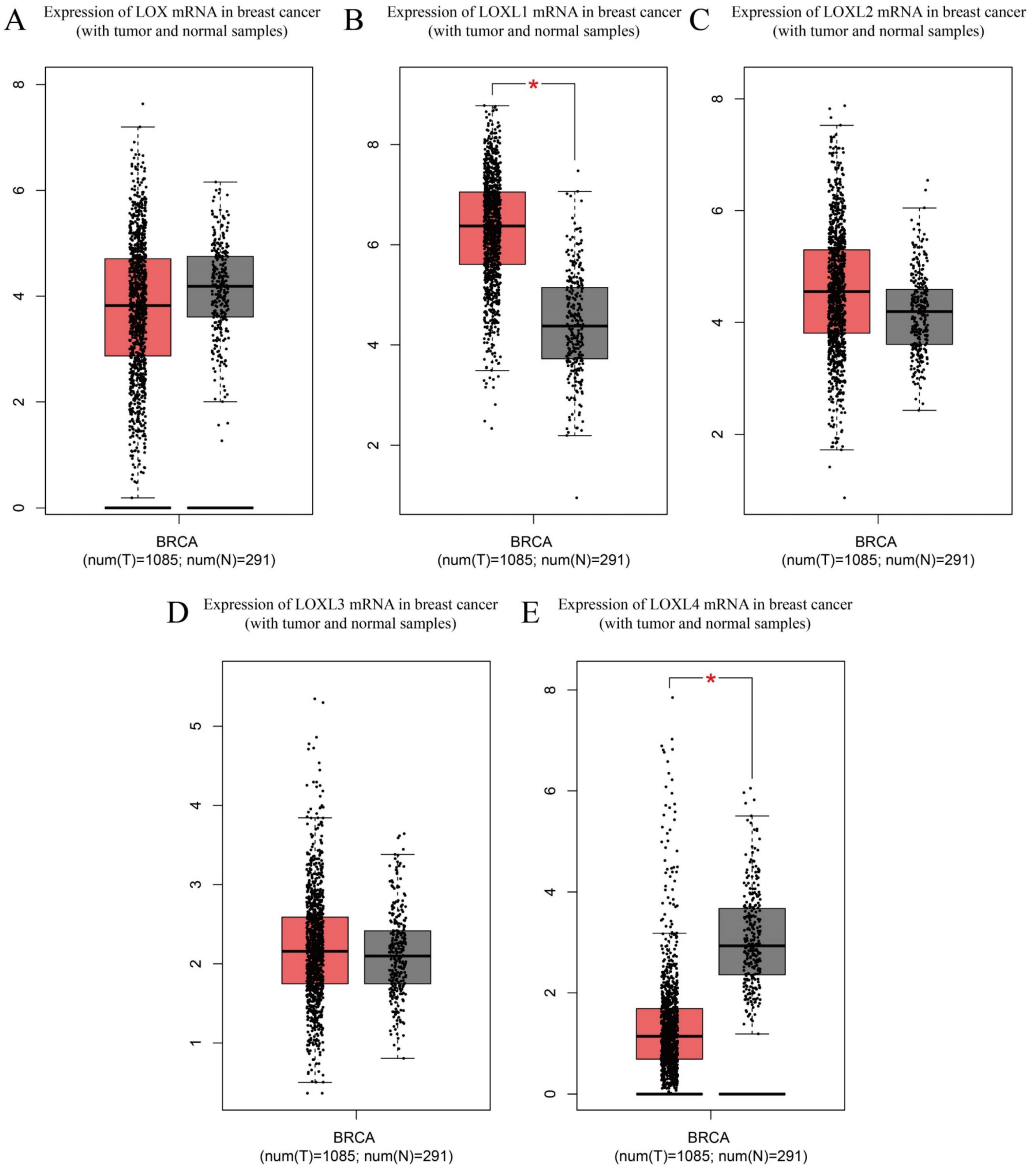
Expression of LOX family mRNAs in breast cancer (with tumour and normal samples) from TCGA database. (A) Expression of LOX mRNA in breast cancers and normal breast samples. (B) Expression of LOXL1 mRNA in breast cancers and normal breast samples. (C) Expression of LOXL2 mRNA in breast cancers and normal breast samples. (D) Expression of LOXL3 mRNA in breast cancers and normal breast samples. (E) Expression of LOXL4 mRNA in breast cancers and normal breast samples. The Cancer Genome Atlas (TCGA); breast cancer (BRCA)

**Figure 3:**
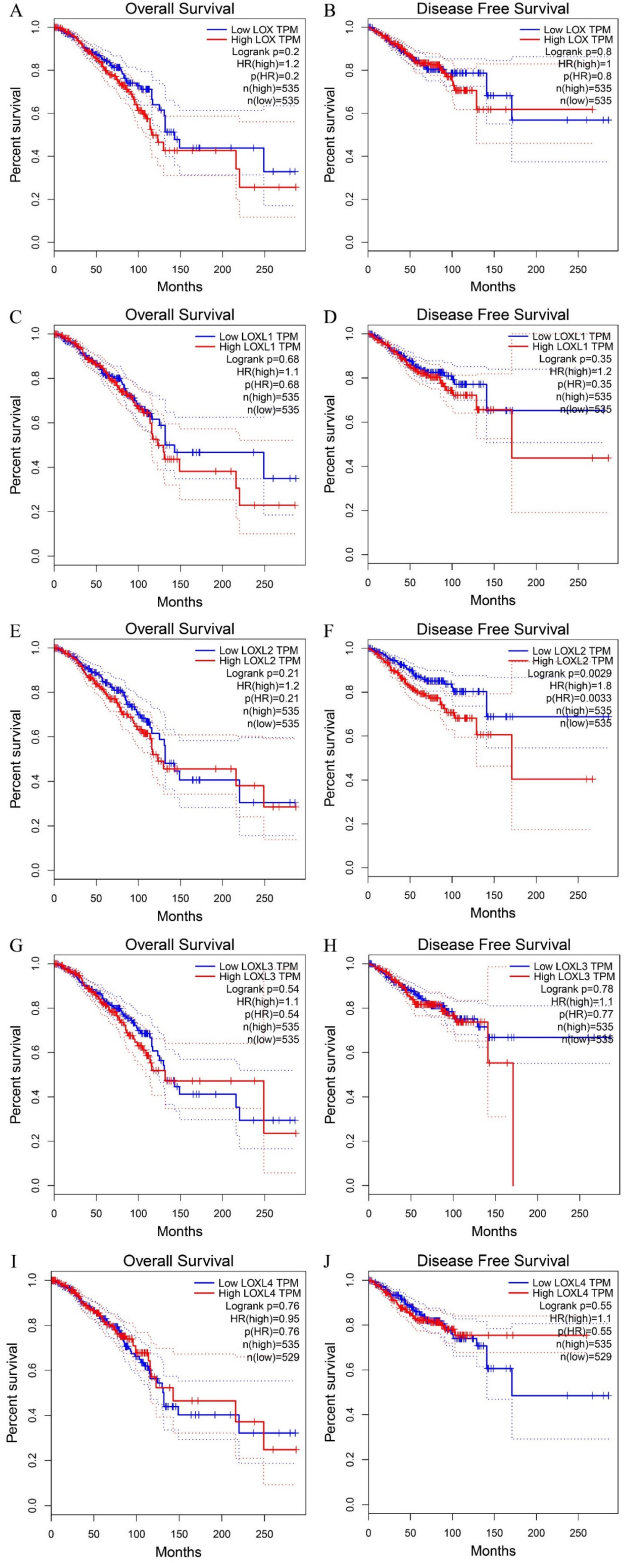
The OS and DFS curve of LOX family proteins in breast cancer. (A) The relationship of the LOX mRNA expression with the OS of breast cancer patients. (B) The relationship of the LOX mRNA expression with the DFS of breast cancer patients. (C) The relationship of the LOXL1 mRNA expression with the OS of breast cancer patients. (D) The relationship of the LOX L1 mRNA expression with the DFS of breast cancer patients. (E) The relationship of the LOXL2 mRNA expression with the OS of breast cancer patients. (F) The relationship of the LOXL2 mRNA expression with the DFS of breast cancer patients. (G) The relationship of the LOXL3 mRNA expression with the OS of breast cancer patients. (H) The relationship of the LOXL3 mRNA expression with the DFS of breast cancer patients. (I) The relationship of the LOXL4 mRNA expression with the OS of breast cancer patients. (J) The relationship of the LOXL4 mRNA expression with the DFS of breast cancer patients. Overall survival (OS); disease free survival (DFS).

LOX family proteins play crucial roles in various biological processes, including the maintenance of normal connective tissue function, embryonic development, and wound healing. In addition to their physiological functions, disruptions in the expression and regulatory pathways of LOX family proteins have been implicated in the onset of various cancers, such as lung cancer 16, hepatocellular carcinoma 17, gastric cancer 18, and colorectal cancer 19. Notably, bioinformatics analysis has identified LOX as a key gene associated with breast cancer metastasis. Studies have indicated that breast cancer patients with heightened levels of LOX expression tend to have notably reduced distant metastasis-free survival (DMFS) 20, highlighting the potential involvement of LOX family proteins in the complex mechanisms driving breast cancer progression and pathogenesis (**Table 2**). According to the analysis of the Cancer Genome Atlas (TCGA) database, LOXL1 is expressed at elevated levels in breast cancer (*p*< 0.05), whereas LOXL4 is expressed at low levels (*p* < 0.05). The expression levels of the other three members of the LOX family in breast cancer are not significantly different (**Figure 2**). With the exception of LOXL2, LOX family members are not significantly associated with the prognosis of patients with breast cancer. Notably, increased LOXL2 expression is significantly linked to a reduced disease-free survival (DFS) period in patients with breast cancer (*p* < 0.01) (**Figure 3**).

### LOX family proteins promote the development of cancer by several mechanisms

The main role of the LOX family is to facilitate the cross-linking of collagen and elastin in the ECM. Recent studies have indicated a strong correlation between LOX family proteins and biological processes, such as cell malignant transformation, tumour cell migration, adhesion, invasion, and the formation of a pre-metastatic microenvironment, and they have reported the crucial involvement of LOX family proteins in tumour initiation and advancement.

#### Promotion of collagen synthesis

Collagen, a key constituent of the extracellular matrix, plays a crucial role in the initiation and progression of tumours. Cells secrete procollagen into the extracellular matrix, which is then converted into collagen through the action of procollagen proteases. The aggregation of collagen is further facilitated by LOX family enzymes. In the hypoxic environment of tumour cells, increased levels of LOX and LOXL2 result in irregular collagen alignment, leading to matrix restructuring. This phenomenon establishes a “highway” for the migration of tumour cells. Overexpression of LOXL2 in noninvasive breast cancer cells induces tumour fibrosis and enhances invasiveness 21. Treatment with neutralizing antibodies against LOXL2 has been shown to significantly reduce the metastatic potential of breast cancer cells 22. Therefore, the regulatory role of the LOX family in collagen cross-linking has emerged as a critical mechanism in promoting tumour metastasis and progression. Further study of its specific mechanisms will aid in the prevention and treatment of cancer.

#### Promotion of epithelial-mesenchymal transition

Various factors can trigger epithelial-mesenchymal transition (EMT) in tumour cells, resulting in decreased cell adhesion and enhanced migratory and invasive capabilities. One such factor, LOX, has been found to induce EMT by downregulating the expression of the E-cadherin epithelial marker and upregulating the transcription of TWIST, a regulatory protein associated with metastasis in breast cancer cells 23, 24. Inhibition of LOX using a specific inhibitor, β-aminopropionitrile, has been shown to hinder hypoxia-induced EMT in cervical cancer cells, underscoring the significant role of LOX in regulating EMT 25. Consequently, the LOX family plays a crucial role in regulating EMT. Nevertheless, the precise mechanisms by which LOX family proteins coordinate EMT remain incompletely understood, thus requiring further in-depth investigation.

#### Activation of the FAK/Src pathway

Focal adhesion kinase (FAK), a tyrosine kinase, is significantly involved in driving tumour progression towards a malignant invasive state. Src, identified as the first proto-oncogene capable of inducing cellular transformation, serves as a central hub in various biochemical pathways upon activation, influencing essential cellular functions, such as adhesion, proliferation, and angiogenesis. The FAK/Src signalling pathway is particularly critical in the context of tumour metastasis. Recent studies have highlighted the impact of LOX in colorectal cancer, where it promotes the proliferation of colorectal cancer cells by modulating Src phosphorylation 26. Similarly, in gastric cancer, LOXL2 has been shown to activate the FAK/Src pathway, thereby facilitating the metastasis of gastric cancer cells 27. As a result, the LOX family may enhance cancer progression by activating the FAK/Src pathway. Further exploration of its specific mechanisms could contribute to cancer prevention and treatment.

#### Promotion of the formation of a pre-metastatic microenvironment

The creation of a favourable microenvironment in the target organ is a crucial step in the metastasis of tumours. Recent research has revealed that LOX, produced by hypoxic tumour cells, plays a significant role in the formation of the premetastatic niche. LOX promotes the growth of metastatic tumours through various mechanisms, including altering the extracellular matrix, attracting CD11b+ bone marrow cells, and facilitating their infiltration into the premetastatic microenvironment 28. In particular, the secretion of LOX by breast cancer cells in the lungs leads to the establishment of a premetastatic niche with bone marrow-derived cells (BMDCs) by restructuring collagen. This process supports the development of lung metastases and subsequent colonization by breast cancer cells 29. Therefore, the LOX family can create a microenvironment conducive to tumour metastasis.

#### Other mechanisms

As research on the LOX family has progressed, a more intricate understanding of its oncogenic mechanisms is being revealed. For instance, LOX has been found to enhance the expression of vascular endothelial growth factor (VEGF) by indirectly activating Akt, also known as protein kinase B (PKB), thereby promoting angiogenesis 30. Moreover, the overexpression of LOX in colorectal cancer cells triggers the production of interleukin-6 (IL-6), which in turn activates the signal transducer and activator of transcription 3 (STAT3) signalling pathway. This series of events fosters receptor activator of nuclear factor kappa-B ligand (RANKL)-dependent osteoclastogenesis and differentiation, ultimately leading to bone metastasis in colorectal cancer 31. LOX has also been shown to inhibit TGF-β1 signalling through the activation of the HTRA1 protease, resulting in the upregulation of the MATN2 protein, which contains an EGF-like domain. The increased expression of MATN2 then initiates EGFR signalling, thereby promoting tumour growth and metastasis 32. Additionally, the N-terminal SRCR sequence of LOXL3 possesses deacetylase activity, which impedes differentiation mediated by STAT3in both normal and cancer cells by reducing STAT3 acetylation 33.

Conversely, studies have suggested that the overexpression of LOXL4 may inhibit human bladder cancer cells by counteracting the Ras/ERK signalling pathway 34. Although the tumour-suppressive function of LOXL4 has been documented in bladder cancer, hepatocellular carcinoma, prostate cancer, and breast cancer, its role in other types of tumours remains ambiguous and warrants further exploration. Consequently, the mechanism by which the LOX family promotes cancer progression is complex, requiring further research to elucidate.

## Role of LOX in breast cancer

The involvement of the LOX protein in breast cancer has been extensively studied. Liu JL *et al.* conducted immunohistochemistry analyses on 111 paired breast cancer and adjacent tissues, as well as 20 benign breast tumour tissues; their findings revealed a significantly greater expression of LOX in breast cancer tissues, with a rate of 48.6% (54/111), compared to adjacent tissues (26.1%; 29/111), and benign lesions (20.0%; 4/20). This elevated LOX expression is associated with larger tumour volumes, advanced clinical stages, lymph node metastasis, and the absence of oestrogen receptor (ER) and progesterone receptor (PR) expression, but it is positively correlated with human epidermal growth factor receptor 2 (HER2) positivity 35. The EMT marker LOX has a differential expression pattern in breast cancer, the strong overexpression of LOX is more prevalent in triple-negative breast cancers than in non-triple-negative breast cancers (p<0.001). Additionally, LOX expression is significantly greater in poorly differentiated (grade 3) breast cancers (p<0.001) 36. Furthermore, LOX is upregulated in metastatic breast cancer cells, promoting cell migration, and modulating cell-matrix adhesion ability through the FAK/Src signalling pathway mediated by hydrogen peroxide 37. Activation of the FAK/Src signaling complex by exogenous expression of mature LOX, but not proLOX, promoted a migratory phenotype through the activation of the p130Cas/Crk/DOCK180 signaling complex and subsequent activation of Rac1 and Cdc42, and inactivation of Rho 38. Thus, mature LOX promoted a migratory phenotype through changes in actin filament polymerization in breast cancer cell lines. One potent downstream signaling molecule that is activated by Src and Rac1 is STAT3 38. Therefore, it is enticing to speculate that LOX can activate STAT3 through Src or Rac1, potentiating a reversal in cellular differentiation and induction of an EMT; however, further experimentation is required to validate this hypothesis.

LOX expression is positively correlated with matrix metalloproteinases (MMPs) and hypoxia-inducible factor 1α (HIF-1α), both of which are linked to breast cancer invasion and metastasis. By using LOX-RNAi-LV to transduce the MDA-MB-231 breast cancer cell line, researchers have observed a significant inhibition in the expression of MMP-2 and MMP-9, leading to reduced invasion and migration abilities of breast cancer cells 35. Moreover, LOX, MMP-2, and MMP-9 are more highly expressed in breast cancer tissues with axillary lymph node metastasis, suggesting a potential synergistic role in breast cancer metastasis. LOX plays a critical role in the metastasis of breast cancer cells and the prognosis of patients. Research has indicated that the overexpression of LOX leads to an increase in collagen fibres, which contributes to lymph node metastasis and heightened tumour rigidity in individuals with invasive breast cancer (IBC) 39. The upregulation of LOX due to chemotherapy in CD8+ T cells has been found to stimulate ECM remodelling in the lungs, thereby facilitating breast cancer metastasis in mice 40. The process of reoxygenation following hypoxia induction is imperative for the catalytic function of LOX, which promotes lung metastasis of breast cancer cells through a hydrogen peroxide-mediated mechanism, particularly in cases associated with an ER-negative status 41. Consequently, the transition from a less aggressive breast cancer to a more aggressive phenotype is driven by hypoxia/reoxygenation, which enhances LOX expression and catalytic activity to some extent 42. Saatci O *et al.* demonstrated that hypoxia triggers an increase in LOX expression via HIF-1α, subsequently activating integrin signalling and leading to decreased survival rates and resistance to chemotherapy in patients with triple-negative breast cancer (TNBC) 43. Additionally, programmed cell death protein-4 (PDCD4) has been shown to decrease LOX expression in a HIF-independent manner, thereby mitigating the migration and invasion of breast cancer cells 44.

**Figure 4:**
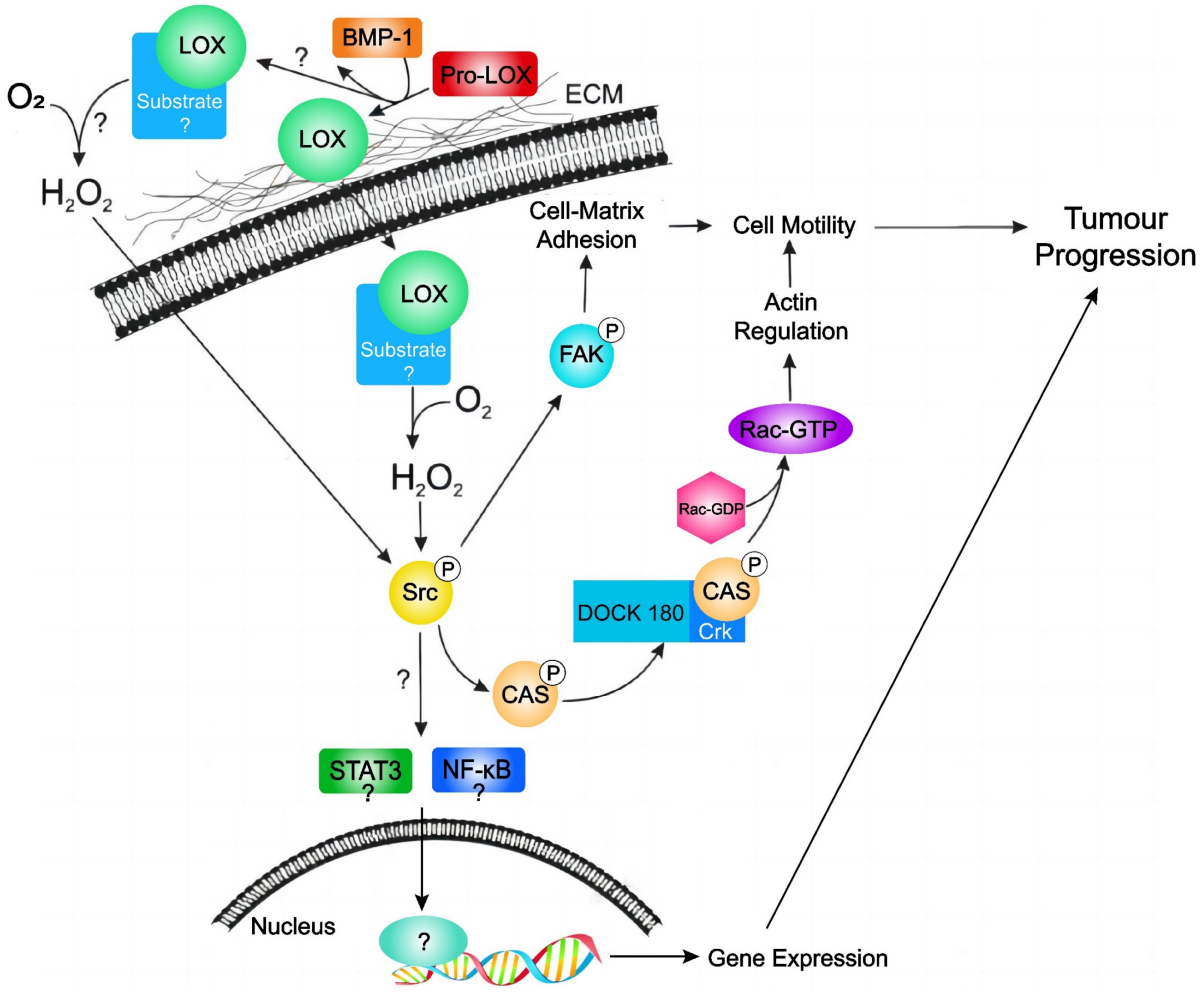
Hypothetical model of LOX activity in promoting tumour progression in breast cancer. LOX is secreted as an inactive proLOX into the ECM where it is cleaved by BMP-1 to become the catalytically active LOX enzyme. Subsequently, active LOX can either translocate into the cell or remain in the ECM. Currently, it is not known where LOX target substrates localize. Subsequent catalytic interaction with substrate produces hydrogen peroxide and stimulates Src activation. Activated Src subsequently activates FAK (leading to changes in cell-matrix adhesion) and/or activates the p130Cas/Crk/DOCK180 signaling pathway (facilitating actin filament formation). Activation of Src may also lead to activation of the STAT3 and NFkB; however, this has not been validated in breast cancer cells. Together, stimulation of these pathways by LOX leads to cell motility and tumour progression in breast cancer. Extracellular matrix (ECM); bone morphogenic protein-1 (BMP-1); focal adhesion kinase (FAK); sarcoma gene (Src); signal transducer and activator of transcription 3 (STAT3); nuclear factor kappa B (NFkB).

In a study focusing on circulating tumour cell colonization and bone metastasis, Cox TR *et al.* showed that LOX regulates the generation of osteoclasts driven by nuclear factor of activated T cell cytoplasmic 1 (NFATc1), which is independent of receptor activator of nuclear factor kappa B (RANK) ligand. This process disrupts normal bone homeostasis and contributes to the formation of osteolytic lesions, bone metastasis, and relapse in patients with ER-negative breast cancer 45. Additionally, Di Mauro P *et al.* reported that the overexpression of LOX (but not LOXL2) in triple-negative breast cancer cells leads to a significant increase in the production of IL-6, a pro-osteoclastic cytokine; they proposed a model suggesting that the combined action of LOX and IL-6, which are secreted by tumour cells, synergistically enhances osteoclast-mediated bone resorption, ultimately promoting the destruction of metastatic bone *in vivo* 46. Individuals with high LOX expression exhibit reduced disease-free survival (DFS) rates, which suggests that increased levels of LOX mRNA expression can be used as a prognostic indicator for worse outcomes in patients with ER-negative breast cancer 47. In summary, LOX is overexpressed in breast cancer, exerts oncogenic effects, and promotes cancer cell mobility and cancer progression by different manners (Figure 4). LOX may also serve as an independent predictor of prognosis in breast cancer patients, highlighting its potential utility in the diagnosis and management of breast cancer.

## Role of LOXL1 in breast cancer

Similar to LOX, lysyl oxidase-like 1 (LOXL1) plays a pivotal role in ECM formation and repair by catalysing the oxidation of lysine residues in elastin and collagen. Sflomos G *et al.* utilized *in situ* hybridization with multiple RNA probes to detect LOXL1 transcript expression in seven samples of invasive lobular carcinoma (ILC) of the breast, and they detected LOXL1 mRNA in all tumour tissues. Immunofluorescence (IF) staining demonstrated the co-localization of ER and LOXL1 in ILC cells, indicating that LOXL1 is expressed by lobular cancer cells in patient tissue samples 48. Knockdown of LOXL1 in MDA-MB-231 and MCF7 breast cancer cells results in a significant decrease in cell proliferation and migration. Additionally, LOXL1 knockdown disrupts ECM remodelling in ILC tumours, attenuats ER signal transduction, and affects tumour growth, invasion, and metastasis *in vivo*, and it is linked to increased malignancy in breast cancer 48. LOXL1 has been found to promote extracellular lactate accumulation under low pH conditions, upregulating MCT1/2 expression, which suggests that LOXL1 plays a role in cancer metastasis under hypoxic conditions 49.

Jeong YJ *et al.* conducted immunohistochemical staining of primary breast cancer tissue and found that the LOXL1 expression level is correlated with intratumoural inflammation and interleukin-4 (IL-4) levels, suggesting a potential immunomodulatory role for LOXL1 50. Using intraductally injected xenografts, Kozma KJ *et al.* reported high LOXL1 expression in ILC breast tumour cells, and they demonstrated that LOXL1 gene knockdown and treatment with the β-aminopropionitrile (BAPN) pan-LOX inhibitor impairs the growth of breast lobule xenograft tumours, reducing tumour cell proliferation and altering collagen fibre tissue 51. Given the toxicity of broad-spectrum lysyl oxidase inhibitors, such as BAPN, future research shoud focus on developing more targeted LOXL1 inhibitors in combination with other clinical therapies to improve the prognosis of ILC patients. As a result, LOXL1 is highly expressed in breast cancer and has carcinogenic effects. Despite substantial evidence supporting the oncogenic role of LOXL1 in breast cancer, further basic experimental research is warranted to elucidate its precise molecular mechanisms.

## Role of LOXL2 in breast cancer

LOXL2 is prevalent across various cancer types. Using a specific ELISA towards the N-terminal neo-epitope site in LOXL2, Leeming DJ *et al.* reported that the serum levels of LOXL2 in healthy individuals average approximately 46.8 ng/mL. Compared to healthy controls, LOXL2 levels are significantly elevated (2.18-fold) in serum from patients with breast cancer 52. Furthermore, the concentrations of LOXL2 in the blood and urine of breast cancer patients are significantly greater than those in healthy controls 53. Research has indicated that LOXL2 expression is typically low in normal breast tissue and primarily localizes within stromal and luminal epithelial cell layers. Conversely, in breast cancer tissue, LOXL2 expression is elevated, and LOXL2 is localized in the cytoplasm, nucleus, and ECM 37. Secreted LOXL2 from breast cancer cells activates stromal fibroblasts in the tumour microenvironment through integrin-induced FAK signalling. This process facilitates ECM remodelling and tumour angiogenesis under hypoxic conditions, thereby promoting breast cancer cell invasion and metastasis 54, 55. Moreover, a significant proportion (60%) of basal-like breast cancer cells exhibit high LOXL2 expression. This protein plays a crucial role in various biological processes, including EMT, as well as the polarity and differentiation of epithelial cells. Nucleus-localized LOXL2 stabilizes the Snail1 transcription factor in the nucleus of breast cancer cells, thereby promoting the EMT process, and it promotes cell invasion more effectively than the secreted LOXL2 protein 54. In particular, LOXL2 modulates the invasive ability of breast cancer cells by regulating the activity of the extracellular proteins tissue inhibitor of metalloproteinases-1 (TIMP1) and MMP-9 56.

Inhibition of LOXL2 through genetic method (knockdown of the LOXL2 gene), chemical intervention (using D-penicillamine), or antibody-based targeting of LOXL2 has been shown to markedly diminish the development of distant metastases in an orthotopic model of immunocompromised breast cancer 37. Intriguingly, these interventions do not impede the growth of primary tumours, underscoring the pivotal role of LOXL2 in the metastatic cascade of breast cancer. High cytoplasmic/perinuclear LOXL2 expression is clearly detected in human basal-like breast cancer (BLBC) patients. This heightened expression significantly augments the likelihood of distant metastasis in breast tumours 57. Weidenfeld K *et al.* demonstrated that dormant tumour cells (DTCs) overexpressing LOXL2 acquire a cancer stem cell (CSC)-like phenotype, promoting the growth and proliferation of recurrent breast cancer cells 58. LOXL2 also increases the expression and secretion of prolymphangiogenic factors in fibroblasts in breast cancer in a HIF-1α-dependent manner, stimulating the Akt-Snail and Erk signalling pathways to enhance lymphangiogenesis and lymph node metastasis in breast tumours 59. LOXL2 is crucial for breast cancer lung metastasis because it promotes dedifferentiation, tumour invasion, and metastasis. Knockdown of the LOXL2 gene has been shown to mitigate lung metastasis in breast cancer cells, whereas overexpression of LOXL2 enhances lung metastasis by upregulating Snail1. This effect has been corroborated in a transgenic mouse model of breast cancer induced by polyomavirus middle T antigen (PyMT) 60.

Research has revealed a mechanism by which LOXL2 facilitates the phosphorylation of ErbB2 (HER2) through the generation of reactive oxygen species (ROS), thereby promoting the progression of atypical hyperplasia and malignant characteristics in normal breast epithelial cells. Moreover, the overexpression of LOXL2 is associated with metastasis in ErbB2-positive breast cancer patients, resulting in decreased MFS and OS rates among affected individuals 61. The degree of LOXL2-mediated H3 oxidation at lysine 4 (H3K4ox) is significantly greater in TNBC MDA-MB-231 cells and patient-derived xenograft tumours than in other breast cancer subtypes. Conversely, the inhibition of LOXL2 expression decreases H3K4ox levels, leading to chromatin decompaction. This process triggers sustained activation of the DNA damage response, thereby increasing the sensitivity of MDA-MB-231 cells to chemotherapy. These changes significantly impact the prognosis of breast cancer patients, highlighting the critical role of LOXL2 in modulating chromatin structure and therapeutic responses in TNBC 62. The SRCR domain of LOXL2 interacts with the N-terminal domain of myristoylated alanine-rich C-kinase substrate-like 1 (MARCKSL1), which inhibits the FAK-Akt-mTOR signalling pathway and MARCKSL1-induced apoptosis, consequently promoting cell proliferation 63.

These results indicate that, similar to LOX, LOXL2 functions as a cancer-promoting protein in breast cancer. Notably, LOXL2 is produced not only by tumour cells but also by stromal cells, highlighting its diverse involvement in the spread of breast cancer. Moreover, LOXL2 has been identified as a potential prognostic indicator for breast cancer, and is capable of distinguishing between different stages of the disease, from carcinoma *in situ* to invasive cancer. Additionally, LOXL2 shows promise in the detection of various phases of breast cancer, from initial to advanced stages. Furthermore, Smithen DA *et al.* demonstrated that the use of the 2-aminomethylene-5-sulfonylimidazole LOXL2 inhibitor significantly reduces the growth of breast cancer tumours in mouse models, resulting in strong antitumour effects 64. Despite these findings, the exact mechanisms underlying the pro-metastatic and invasive functions of LOXL2 in breast cancer are not fully understood. Nevertheless, research on LOXL2 has suggested that both its intracellular and extracellular forms play a role in the progression of breast cancer. In conclusion, LOXL2 is highly expressed in breast cancer, serving as a potential prognostic indicator, and promoting breast cancer cell growth, proliferation, migration, and invasion. Consequently, high LOXL2 expression is considered a significant risk factor for the early development of breast cancer metastasis.

## Role of LOXL3 in breast cancer

Limited research has been conducted on the function and mechanism of LOXL3 protein in breast cancer. This enzyme was initially identified as a lysyl oxidase owing to its sequence resemblance to other members of the LOX family. By immunohistochemical analysis of cancer tissues from 291 invasive breast cancer patients, Jeong YJ *et al.* investigated the correlation between LOXL3 expression and clinicopathological parameters, and they reported significant relationships of LOXL3 expression with ER expression, PR expression, and breast cancer molecular subtypes (*P*<0.001) 50. In addition, Sebban S *et al.* detected LOXL3 protein expression in primary tumour tissue and pleural effusion of breast cancer patients via immunohistochemical staining of primary breast cancer tissue, which indicated a significant correlation of the expression of the LOX and LOXL3 genes with intra- and para-cancerous inflammation in breast cancer 65. Koorman T *et al.* utilized immunofluorescence techniques to explore the localization of LOXL3, and they reported predominant expression of LOXL3 in surrounding myoepithelial cells in normal human breast tissue and invasive ductal carcinoma no special type (IDCNST), with minimal expression in the cancer cells themselves, highlighting the role of LOXL3 in promoting the collective invasion of ductal breast cancer cells into the collagen matrix through its involvement in collagen remodelling processes. Subsequent analysis of a clinical tissue microarray (TMA) comprising 368 invasive breast cancer samples has revealed cytoplasmic expression of LOXL3 in approximately 26.0% (76/292) of breast cancer patients. Despite the specific expression of LOXL3 by mammary gland myoepithelial cells and the focal upregulation of LOXL3 in CK14-positive cells in contact with collagen, no association has been identified between LOXL3 expression and triple-negative breast cancer. Although LOXL3 is expressed in high-grade invasive breast cancer, no correlation has been observed between LOXL3 expression and tumour size, mitotic index, or lymph node status 66.

Research has underscored the significance of LOXL3 expression in various malignancies, including hepatocellular carcinoma, pancreatic cancer, glioma, and primary melanoma. Therefore, we speculate that LOXL3 may play a role in promoting breast cancer. However, there are few studies on the role of LOXL3 in breast cancer. Additional research is warranted to clarify the association between LOXL3 and breast cancer, as well as its fundamental mechanisms.

## Role of LOXL4 in breast cancer

There is limited research on LOXL4, the final member of the LOX protein family to be identified and characterized, in human malignant tumours. Previous studies have suggested that increased levels of LOXL4 are linked to increased tumour proliferation or migration in various cancers, such as liver cancer, head and neck squamous cell carcinoma, oesophageal cancer, gastric cancer, and colorectal cancer. However, in lung cancer and bladder cancer, LOXL4 may function as a tumour suppressor, as LOXL4 deficiency enhances cancer cell proliferation and metastasis 67. Consequently, the role of LOXL4 in promoting or inhibiting human malignant tumours appears to be contingent on the specific tumour cell environment and the stage of tumour advancement.

In contrast to that of LOXL1, LOXL2, and LOXL3, the expression of LOXL4 mRNA is consistently reduced across all subtypes of breast cancer, including basal-like, HER2+, luminal A, and luminal B subtypes, in comparison to that in normal breast tissue 20. Wuest M *et al.* conducted an analysis of 56 TNBC and 112 ER+ breast cancer samples, and they revealed elevated levels of LOXL4 and HIF-1α mRNA in the cancer tissues of TNBC patients compared with those of ER+ breast cancer patients 68. Notably, the alternative splicing of LOXL4 results in specific splicing variants primarily detected in effusion specimens from breast cancer patients but not in primary tumour tissues 69. These variants significantly impact cancer cell metastasis *in vitro* and tumour progression *in vivo* in breast cancer patients. In an orthotopic xenograft mouse model of TNBC, suppression of the LOXL4 gene enhances primary tumour growth and lung metastasis, underscoring the involvement of LOXL4 in the invasive progression of TNBC 70.

Decreased LOXL4 expression initiates ECM remodelling, triggering collagen production, accumulation, and subsequent structural changes. Suppression of LOXL4 has been demonstrated to increase the levels of type I and type IV collagen, resulting in the thickening of collagen fibres in transplanted TNBC tissues. These modifications have been associated with the promotion of tumour growth and metastasis, which are indicative of a worse prognosis for TNBC patients 70. The combination of low LOXL4 expression and high collagen levels has been correlated with decreased hazard ratios (HRs) for both OS and DFS in breast cancer patients, particularly those with the HER2+ subtype. Yin H *et al.*, introduced of MDA-MB-231-LOXL4 cells into nude mice via subcutaneous and tail vein injections, which led to larger tumour sizes and notable lung metastases. In addition, treatment with BAPN to inhibit LOXL4 enzyme activity resulted in a significant decrease in breast tumour size and weight, while the suppression of LOXL4 gene expression through miRNA markedly reduced the proliferation, migration, and lung metastasis of breast cancer cells both *in vitro* and *in vivo* 71. These findings suggest that LOXL4 may exhibit high expression in breast cancer. Developing small molecule inhibitors targeting LOXL4 could provide promising therapeutic options for breast cancer. This underscores the crucial role of LOXL4 in driving the onset and progression of breast cancer.

## Conclusions and Future Perspectives

In recent years, advancements in medical standards and the identification of multiple biomarkers have significantly improved the accuracy of diagnosing and predicting outcomes for breast cancer patients. Despite these advancements, the development of distant metastasis remains a key factor affecting survival rates in individuals with breast cancer 72. Recent research has suggested that LOX family proteins play a crucial role in promoting cancer, especially in regulating key processes involved in the initiation and spread of breast cancer. These proteins not only enhance tumour metastasis within the tumour microenvironment but also mediate intracellular EMT remodelling. LOX family proteins facilitate the movement and invasion of breast cancer cells, and are closely associated with poor prognosis in affected patients. Previous studies have highlighted the clinical importance of the LOX family of proteins, indicating their potential usefulness in various aspects of breast cancer management. These proteins show promise in facilitating early breast cancer detection and may serve as valuable biomarkers for determining disease stage, grade, and metastasis, as well as for assessing chemotherapy effectiveness in breast cancer patients. Notably, LOX and the LOXL2 to LOXL4 isoforms have been identified as potential targets for preventing breast cancer metastasis. Ongoing efforts to develop inhibitors have resulted in the use of various compounds, including LOXL2 inhibitors, dual inhibitors targeting both LOX and LOXL2, dual inhibitors of LOXL2 and LOXL3, and pan-LOX inhibitors. These inhibitors exhibit varying levels of selectivity for different enzymes within the LOX family, highlighting the necessity for a nuanced approach in therapeutic interventions. As research advances, exploring novel drug therapies targeting LOX family proteins holds great promise for advancing personalized and precise treatment strategies for breast cancer patients. Additionally, considering the potential role of LOX family proteins in modulating the tumour immune microenvironment, an area that is currently underexplored, investigating the regulatory mechanisms of LOX family proteins on the immune microenvironment of breast cancer and exploring the synergistic effects of LOX inhibitors with immunotherapy present compelling avenues for future research. These efforts have the potential to enhance the understanding of the intricate interplay between LOX family proteins and the immune system in breast cancer, providing new possibilities for innovative therapeutic approaches that may significantly improve patient outcomes.

To obtain a thorough understanding of the roles played by LOX family proteins in the initiation and progression of breast cancer, it is essential to address several key issues. First, there is a lack of research exploring the oncogenic effects of LOXL1 and LOXL3 in breast cancer. Further fundamental and clinical investigations are necessary to establish a robust foundation concerning the participation of LOXL1 and LOXL3 in the progression of breast cancer. Second, existing studies have primarily concentrated on identifying downstream targets of LOX family proteins. Nevertheless, the regulation of these proteins by upstream genes and their degradation pathways remain inadequately understood. The generation of gene knockout cells, knock-in cells, or mouse models will facilitate a more comprehensive examination of these regulatory mechanisms. Given the complex protein structure and diverse functions of LOX family members, it is plausible that they engage in interaction networks with other proteins through multiple domains to coordinate various biological functions. Identification of effective biomarkers for LOX family proteins, clarification of how these proteins bind to substrates in breast cancer, and elucidation of the molecular mechanisms involved will be crucial. Furthermore, determining the optimal timing for intervention to achieve maximal efficacy is a promising direction for future research. It is important to note that drugs developed to target LOX family members have been effective at inhibiting the progression of breast cancer in preclinical models, and have shown efficacy in clinical trials of other cancer types. Investigations into miRs-dictated mechanisms for the activity of LOX family members could further shed light on the molecular activity of TME and pave the way to prospective clinical therapeutic approaches. To summarize, LOX family members represent attractive therapeutic targets for the treatment of breast cancer.

## Funding

This research was funded by the National Natural Science Foundation of China (Nos. 32160167 and 82160516). Yunnan Provincial Applied Basic Research Program General Project (Nos. 202201AT070004 and 202301AT070023). Key Program of Yunnan Provincial Applied Basic Research Program (No. 202001BB0500080). Yunnan Province Ten Thousand Talent Program (2019). Yunnan Provincial Local University Joint Project-Youth Project (No. 202101BA070001-282). Scientific Research Fund of Yunnan Provincial Department of Education (Nos. 2022J0688, 2022J0716 and 2022Y808). The Dali City Science and Technology Planning Project Support (No. 2021KBG032).

## Figures and Tables

**Table 1:** LOX family: genomic and protein organization

Gene	Chromosome location	Exon count	Number of amino acids	Molecular weight (kD)	Catalytic activity (inhibitable by BAPN)
LOX	5q23.1	8	417	32	+
LOXL1	15q24.1	9	574	63	+
LOXL2	8p21.3	14	774	87	+ (not inhibitable by BAPN)
LOXL3	2p13.1	16	753	80	+
LOXL4	10q24.2	17	756	82	+

Note: BAPN, β-aminopropionitrile

**Table 2:** Role of LOX family proteins in progression and prognosis of breast cancer

LOX family members	Leading role	Relationship with prognosis	Downstream gene targets	References
*LOX*	Oncoprotein, promote tumour invasion and migration	High expression of LOX can be used as an indicator of poor prognosis in ER- breast cancer patients	MMP-2, MMP-9	35, 41, 43, 45, 47
*LOXL1*	Oncoprotein, promote tumour proliferation, invasion and migration	High expression of LOXL1 as an indicator of poor prognosis in breast cancer	N/A	48, 49, 51
*LOXL2*	Oncoprotein, promote tumourigenesis, invasion and migration	High LOXL2 expression is associated with poor survival in ER- breast cancer patients, resulting in poor MFS and OS in breast cancer patients	TIMP1, MMP-9	37, 52, 54, 59, 62
*LOXL3*	Oncoprotein, may promote tumourigenesis	LOXL3 positive can be used as an indicator of poor prognosis in patients with breast cancer	N/A	50, 65, 66
*LOXL4*	Oncoprotein, promote tumourigenesis and progression	Low LOXL4 and high collagen expression are associated with poor prognosis in breast cancer patients, with the strongest association in TNBC patients	COL I, COL IV	68, 70, 71

Note: MMP-2, matrix metalloproteinase-2; MMP-9, matrix metalloproteinase-9; TIMP1, tissue inhibitors of metalloproteinases-1; COL I, Collagen Type I; COL IV, Collagen Type IV; N/A, not available.
